# C677T gene polymorphism of *MTHFR* and metabolic syndrome: response to dietary intervention

**DOI:** 10.1186/s12967-014-0329-4

**Published:** 2014-11-29

**Authors:** Laura Di Renzo, Luigi Tonino Marsella, Francesca Sarlo, Laura Soldati, Santo Gratteri, Ludovico Abenavoli, Antonino De Lorenzo

**Affiliations:** Division of Clinical Nutrition and Nutrigenomics, Department of Biomedicine and Prevention, University of Rome “Tor Vergata”, Rome, 00133 Italy; Division of Legal Medicine and Social Security, Department of Biomedicine and Prevention, University of Rome “Tor Vergata”, Rome, 00133 Italy; Department of Agriculture, University of Naples “Federico II”, Portici, 80055 (Na) Italy; Department of Health Sciences, University of Milan, Milan, Italy; Department of Surgery and Medical Science, University “Magna Græcia”, Germaneto, (CZ) 88100 Italy; Department of Health Science, University “Magna Græcia”, Germaneto, (CZ) 88100 Italy; Clinic “Nuova Annunziatella”, Rome, 00147 Italy; I.N.Di.M, National Institute for Mediterranean Diet and Nutrigenomic, Amantea, (CS) 87032 Italy

**Keywords:** MTHFR C677T gene polymorphism, Body composition, Metabolic syndrome, Hypocaloric balanced diet

## Abstract

**Background:**

Methylenetetrahydrofolate reductase (MTHFR) gene polymorphisms were found associated with body mass index (BMI)-defined obesity and lean mass.

The aim of our study was to examine the role of the C677T *MTHFR* gene polymorphism in the response to diet in the management of metabolic syndrome. We investigated the body composition and metabolic factor changes after an hysocaloric balanced diet (HBD), in Italian obese women affected by metabolic syndrome (MS).

**Methods:**

Forty four obese women affected by MS were eligible for the study. A HBD for 12 weeks was assigned. Study participation included a complete screening for dietary habits, anthropometry, body composition, blood biochemical markers and C677T MTHFR polymorphism genotyping. The study has been registrated by ClinicalTrials.gov Id: NCT01890070.

**Results:**

The highest number of responders to HBD nutritional intervention were T(−) carriers (p ≤ 0.05). In the 81% of the total population a loss of Total Body Lean was observed. A significative loss (p ≤ 0.05) of Total Body Lean was observed in the 47% of T(−) carriers and in the 53% of T(+) carriers. Diastolic and systolic blood pressure, and waist circumference were reduced (p ≤ 0.05). The prevalence of MS parameters decreased by 84% for systolic and diastolic blood pressure; 79,5% for HDL cholesterol, 82% for fasting glucose and 77% for triglycerides.

**Conclusions:**

MTHFR genetic variations analysis would be an innovative tool for the nutritional assessment. Our data provide the basis for personalized dietary recommendations based on the individual’s genetic makeup and nutritional status.

**Trial registration:**

The study has been registrated by ClinicalTrials.gov Id: NCT01890070.

## Introduction

According to the definition of World Health Organization WHO, obesity is a condition char-acterised by a large increase of body fat [1]. Body weight and its component are related to energy expenditure and metabolic risk. Pathogenic events related to changes in fat mass are basis of the obesity: fat amount and its distribution is a critical value. In particular, high fat mass has been associated to increased risk of chronic condition, such as insulin resistance, and cardiovascular disease (CVD) [2]. Therefore, high-risk management strategies remain important as long as the disease is widely prevalent, and intervention to target population level behavior is critically needed. To this aim, the clustering of certain metabolic factors (e.g., abdominal obesity, dyslipidemia and elevated blood pressure), that define the metabolic syndrome (MS), has been documented to identify patients at increased risk for type 2 diabetes, as well as CVD and overall mortality [3,4]. The MS is a growing health problem worldwide with an estimated prevalence in developed countries of 25–35% in adults, in parallel with the increasing prevalence of obesity and diabetes. [5]. A similar increase has also involved the Mediterranean population [6], due to diet changes, excess weight, central obesity, and sedentary way of life.

Beyond metabolic factors, the genetic background may affect the risk of cardiometabolic diseases, expecially in women in the post-menopausal period. Indeed, there are data suggesting the existence of significant gene-environment interaction effects that make some individuals more susceptible to body weight gain or loss than others because of genetic differences, when exposed to an obesogenic environment. Moreover, body composition changes could be partially explained by genetic factors or by the interaction between genes and environment [7,8].

Personalized risk prediction and strategic health-care planning will facilitate a new form of care, which is called ‘prospective health care’ [9]. However, in order to provide an individual and personalized health plan, new tools and predictive biomarkers are needed.

Methylenetetrahydrofolate reductase (MTHFR), by catalyzing the conversion of 5, 10 methylenetetrahydrofolate to 5-methyltetrahydrofolate, is a pivotal enzyme in folate metabolism that regulates the proportional usage of one-carbon units between methylation reactions and nucleic acid synthesis. The C677T MTHFR gene polymorphism results in a reduced specific MTHFR activity (~34% residual activity in T677T, ~71% residual activity in C677T relative to C677C).

The MTHFR gene polymorphisms were also found to be associated with body mass index (BMI)-defined obesity and lean mass, sustaining a linkage of body mass index (BMI) and lean mass to chromosome 1p36, where the MTHFR gene is located [10,11].

However, weight loss and the body mass index (BMI) lack sensitivity to detect total body lean mass (TBLean) loss. Moreover, BMI alone may not be enough accurate for assessing the extent of excessive fat accumulation and thus the obesity related disease risk, because it does not directly measure the total body fat mass (TBFat). Body composition evaluation is a valuable technique to assess nutritional status, because it gives a precise evaluation of nutritional status through the assessment of TBFat, TBLean, and bone mass, allowing evaluation of the disease prognosis and outcome [12], providing more reliable and precise measure for obesity [13,14]. Dual energy X-ray absorptiometry (DXA) can measure body composition, and DXA-derived TBLean measures can reflect both muscle mass and muscle strength [15], providing a reliable measure for assessment of sarcopenia [16], and obesity [17].

Furthermore, we have shown a significant relationship between the Normal Weight Obese phenotype, a status of TBFat accumulation accompanied by TBLean deficiency, and possession of wild type and heterozygous genotypes of C677T MTHFR enzyme polymorphism [18]. These results jointly imply that MTHFR may play a role in variation of TBLean.

Due to recent lines of evidence supporting the functional and genetic relevance of C677T MTHFR gene polymorphism, this study was designed to determine the role of this polymorphism in the response to hysocaloric balanced diet (HBD), in terms of weight lost, body composition, blood pressure, and gluco-lipid profile, in Italian women with MS.

## Methods

### Study design and subjects

We used intervention study design where each subject acts as their own control group with repeated measures, at baseline and after 12 weeks nutritional intervention period.

A total of 50 participants were consecutively recruted by Clinical Nutrition and Nutrigenomic Division of University of Rome Tor Vergata during three months from May 2013. Participation in the study included a complete medical history to gather informations about health status, current medications history, including supplements of vitamins and minerals, social habits, like alcohol drinking and smoking, food habits, physical activity and family history for chronic diseases, a complete screening of anthropometry and body composition, and a genotyping for the C677T MTHFR polymorphism. Subjects having serious chronic diseases/conditions that may have potential influence on endocrine and metabolism were excluded

The study group consisted of 44 obese women affected by MS. The subjects were classified as obese, according to WHO criteria [19], and percentage of total body fat (PBF) cutoff points [13].

A multidisciplinary team of dieticians and nutritionists met with each patient, and provided an educational session for nutrition and meal-planning guidance.

A balanced hysocaloric diet for 12 weeks was assigned to all eligible subjects. None of the subjects was receiving drug treatments at the time of the assessment.

A statement of informed consent was signed by all participants in accordance with principles of the Declaration of Helsinki. Nutritional status assessment was conducted at the Obesity Centre of Nicotera Hospital (VV, Italy). Genetic analysis was performed at the Clinical Nutrition and Nutrigenomic Section, Department of Biomedicine and Prevention of University of Rome Tor Vergata.

### Physical Activity (PA) assessment

Leisure time and PA habits were monitored using a validated questionnaire [20]. No change of total energy expenditure (kcal/day) was required during the experimental time. Participants were asked to maintain their usual exercise habits during 3 months of follow-up after the beginning of the nutritional intervention.

### Nutritional intervention

The macronutrient’s composition of the HBD, [21] was as follows: 55% of kcal/day from carbohydrates; 15% of kcal/day from proteins; 30% of kcal/day from lipids (saturated fat ≤10%; poliunsaturated fatty acids (PUFA) 6–10%: 5–6% of n-6 PUFA and 1–2% of n-3 PUFA; monounsaturated fatty acids (MUFA), about 15%; trans fatty acids ≤1%).

The HBD was performed as hypocaloric, respect to the dietary habitus, taking into account the resting metabolic rate (RMR) of each subjects, calculated using De Lorenzo et al. prediction equation for the Italian population [22], and level of PA [20].

### Anthropometric measurements

After a 12-h overnight fast, all subjects underwent anthropometric evaluation. Anthropometric parameters of all the participants were measured according to standard methods (body weight, height, waist and hip circumferences) [23]. Subjects were instructed to take off their clothes and shoes before performing all the measurements. Body weight (Kg) was measured to the nearest 0.1 Kg, using a balance scale (Invernizzi, Rome, Italy). Height (cm) was measured using a stadiometry to the nearest 0.1 cm (Invernizzi, Rome, Italy). The waist (WC) and hip (HC) circumferences were measured with a flexible steel metric tape to the nearest 0.5 cm. WC was measured at the horizontal plane that corresponds with the narrowest point between the crest iliac and the bottom rib. HC was measured at the largest point when observed on a horizontal plane. BMI was calculated using the formula: BMI = body weight (Kg)/height (m)^2^.

### Dual-energy X-ray absorptiometry (DXA)

Body composition analysis was assessed by DXA (i-DXA, GE Medical Systems, Milwaukee, WI, USA), according to the previously described procedure, for evaluating soft tissues, i.e. TBFat and TBLean [24,25]. The subjects were instructed not to exercise within 24 h from the test. The subjects were given complete instructions on the testing procedure. They wore a standard cotton t-shirt, shorts and socks. They laid supin on the DXA, without moving while the DXA scan recorded their results. The average measurement time was 20 min. Radiation exposure was equivalent to 0.01 mSv. The coefficient of variation (CV% =100 × s.d./mean) intra- and intersubjects ranged from 1 to 5%. The coefficient of variation for bone mass measurements is ≤1%; coefficient on this instrument for five subjects scanned six times over a 9-month period were 2.2% for TBFat and 1.1% for TBLean.

The resting metabolic rate (RMR) of each subjects, was calculated using De Lorenzo et al. prediction equation for the Italian population, and level of PA [22].

### Clinical and metabolic variables

The blood pressure was taken using a manual mercury sphygmomanometer from the right upper arm after the subject was seated quietly for at least 5 min (average of three measurements).

Blood samples (10 mL) were collected into sterile tubes containing EDTA (Vacutainer®). All materials were immediately placed on ice and plasma was separated by centrifugation at 1600 × g for 10 min at 4°C.

Serum laboratory tests included fasting glucose, HDL-cholesterol and Triglycerides at baseline and after 12 weeks of HBD. Fasting glucose concentrations were measured using the glucose oxidase method with an automated glucose analyzer (COBAS INTEGRA 400, Roche Diagnostics, Indianapolis, IN, USA), serum lipid profile components were determined by standard enzymatic colorimetric techniques (Roche143 Modular P800, Roche Diagnostics, Indianapolis, IN, USA).

Analyses were carried out by the accredited Clinical Chemical Laboratories of the Hospital of Nicotera, Italy.

The Diagnosis of MS was based on the International Diabetes Federation (IDF) guidelines [26]. Diagnosis required central obesity, defined as waist circumference ≥80 cm for European women, plus any two of the following four factors: TG level ≥150 mg/dL or specific treatment for this lipid abnormality; HDL cholesterol ≤40 mg/dL in males and ≤50 mg/dL in females or specific treatment for this lipid abnormality; systolic blood pressure (SBP) ≥130 mm Hg or diastolic blood pressure (DBP) ≥85 mm Hg or anti-hypertensive treatment; fasting plasma glucose ≥100 mg/dL or previously diagnosed type 2 diabetes.

### MTHFR genotype analysis

DNA was isolated from peripheral leukocytes by using the Gentra DNA isolation kit (Gentra Systems Inc, Minneapolis, MN, USA). According to a previously described procedure, genotyping for the MTHFR point polymorphism C677T was performed by polymerase chain reaction amplification with the primers 5′TGAAGGAGAAGGTGTCTGCGGGA3′ (sense) and 5′AGGACGGTGCGGTGAGAGTG3′ (antisense). Thirty cycles (95°C for 45 s, 64°C for 30 s, and 72°C for 30 s) were used to amplify the 198-base pair (bp) product. Because the C-to-T transition at nucleotide 677 produces a HinfI digestion site, the amplified product derived from the mutant gene was cleaved into 175-bp and 23-bp fragments by HinfI, which leaves the wild-type gene unaffected. After electrophoresis through a 6% polyacrylamide gel, the digestion products were visualized by staining with ethidium bromide.

### Statistical analysis

Data are presented as group means (or median) ± standard deviation (SD), percentage, or ≤ DELTA% > (Δ%). Δ% expresses the relative change, in percent, of a parameter respect to baseline. For the calculation we used the following formula:

Δ% = [(value at week 12) – (value at baseline)/(value at baseline)] ∗100.

Sample size was selected to provide a statistical power of 95% to detect a reduction of 50% for the MS, with alpha =0.05.

Data were analyzed to check assumptions about the distribution of the measured variables. Three genotype groups were first considered to check differences in considered variables between groups. Because a dominant or recessive effect existed, analysis was repeated comparing carriers T(+) *vs* non-carriers T(−) groups. An independent t test were performed to evaluate differences before and after HBD nutritional intervention. The probability values are 2-sided; a probability value ≤0.05 was considered to indicate statistical significance. Statistical analysis was performed using a computer software package (SPSS for Windows, version 13.0; SPSS Inc., Chicago, IL, USA).

## Results

### Dietary and physical assessment

Of all recruited women, 44 of them (88%, age: 50.20 ± 10.12 years) completed the study, due to the lost of follow-up of 6 subjects, and their results were eligible for data analysis. According to WHO criteria of obesity, all subjects were obese with a BMI between 56.23 kg/m^2^ and 33.37 kg/m^2^ at the starting point, before the nutritional intervention.

Dietary assessment is reported in Table 1. A comparison of macronutrients and micronutrients between baseline and HBD nutritional intervention is highlighted.

**Table 1:** **Dietary assessment at baseline and after HBD nutritional intervention** ^**1**^

	**Baseline**	**HBD nutritional intervention**
**Nutrients**		
**Energy (Kcal/day)**	2950 ± 390	2410 ± 215
**Carbohydrates (% en)**	48 ± 2	53 ± 1
**Lipids (% en)**	35 ± 1	26 ± 1
**Proteins (% en)**	13 ± 1	17 ± 1
**Fiber (g/die)**	18 ± 4	33 ± 2
**Folate (μg/die)**	275 ± 50	466 ± 20
**Vitamin B12 (μg/die)**	3 ± 1	5 ± 1
**Vitamin B6 (μg/die)**	2 ± 1	2 ± 1

^1^Values expressed as mean ± SD.

The level of PA was classified into three categories (sedentary, moderate and vigorous) based on the time spent on life activity or programmed physical exercise. According to PA questionnaire, at baseline, no subjects spent time for vigorous PA, the most spent 1–2 times/week for moderate PA, with a sedentary behavior ranging from 10 to 16 h/day. Nobody changed the level of PA during the HBD period.

### Genotyping assessment

According conventional formula of the Hardy-Weinberg rule, the *T* allele frequency was 2.13%, and the *CC*, *CT*, and *TT* genotype frequencies were 62%, 33.5%, and 4.5%, respectively. The *T* allele frequency in our sample was similar to that in other white populations. The study population was divided in 2 subgroups, i.e. T(+) carriers and T(−) non-carriers of the C677T MTHFR gene polymorphism.

The T(+) and T(−) frequency was 54.5% (*n* =24), and 45.5% (*n* =20), respectively.

### MS components

MS componenets at baseline and after HBD, for total population and according to MTHFR genotypes, are given in Table 2.

**Table 2:** **Comparison of clinical parameters of metabolic syndrome at baseline and after HBD nutritional intervention** ^**1**^

**Parameters**	**Baseline**			**Week 12**		
	**Total (n = 44)**	**T (−) (n = 20)**	**T (+) (n = 24)**	**Total (n = 44)**	**T (−) (n = 20)**	**T (+) (n = 24)**
**Waist (cm)**	111.83 ± 11.65	106.06 ± 10.63	116.17 ± 10.80^d^	103.50 ± 13.07^a^	97.50 ± 12.74^b^	108.00 ± 11.87^c^
**SBP (mmHg)**	131.67 ± 12.96	135.56 ± 13.33	128.75 ± 12.43	121.05 ± 10.12^a^	119.67 ± 12.54^b^	122.08 ± 10.33
**DBP (mmHg)**	84.33 ± 9.45	84.44 ± 11.30	84.25 ± 8.32	75.48 ± 6.10^a^	75.56 ± 7.26	75.42 ± 5.42^c^
**Fasting Glucose (mg/dl)**	109.68 ± 22.33	115.33 ± 25.72	104.60 ± 18.66^d^	97.24 ± 7.83	93.57. ± 7.74^b^	99.80 ± 7.16^e^
**HDL-C (mg/dl)**	44.88 ± 11.67	46.59 ± 11.79	43.55 ± 9.58	47.40 ± 15.50	49.15 ± 7.30	46.22 ± 10.78
**Triglycerides (mg/dl)**	171.11 ± 71.49	164.33 ± 64.31	177.90 ± 79.50^d^	166.47 ± 64.94	142.62 ± 59.04^b^	173.57 ± 63.28

^1^All values are mean ± SD. SBP: systolic blood pressure; DBP: diastolic blood pressure; HDL-C: HDL cholesterol.

^a^Reflects the significance of the differences between total population at baseline vs total population at week 12 determined with an independent t test (p ≤ 0.05).

^b^Reflects the significance of the differences between T(−) carrier at baseline vs T(−) carrier at week 12 determined with an independent t test (p ≤ 0.05).

^**c**^Reflects the significance of the differences between T(+) carrier at baseline vs T(+) carrier at week 12 determined with an independent t test (p ≤ 0.05).

^d^Reflects the significance of the differences between T(−) carrier at baseline vs T(+) carrier at baseline determined with an independent t test (p ≤ 0.05).

^e^Reflects the significance of the differences between T(−) carrier at week 12 vs T(+) carrier at week 12 determined with an independent t test (p ≤ 0.05).

In the total population, the WC, SBP, DBP showed the most changes (p ≤ 0.05). After HBD nutritional intervention, a significant decreased level (p ≤ 0.05) of fasting blood glucose was observed in T(−) carries levels. Moreover, in T(+) and T(−) carriers significant decreased level of triglycerides was highlighted (p ≤ 0.05).

Moreover, 57% of T(+) carrier did not ameliorated SBP and DBP, 56% T(+) carrier did not ameliorated HDL-C, 75% T(+) carrier did not ameliorated fasting glucose, and 60% T(+) carrier did not ameliorated triglycerides values.

### Anthropometric and body composition parameters

Table 3 shows the distribution of obese subject according to obesity degree at baseline and after HBD.

**Table 3:** **Distribution of obese subjects according to obesity degree at baseline and after HBD nutritional intervention** ^**1**^

**Obesity (%)**	**Total (n = 44)**			**T (−) carrier (n = 20)**			**T (+) carrier (n = 24)**		
	**Baseline**	**Week 12**	**∆**	**Baseline**	**Week 12**	**∆**	**Baseline**	**Week 12**	**∆**
**I degree (BMI 30–35 Kg/m** ^**2**^ **)**	23.81	47.62	+ 23.81	44.44	66.67	+ 22.23	8.33	33.33	+ 25
**II degree (BMI 35–40 Kg/m** ^**2**^ **)**	33.33	23.81	- 9.52	33.33	22.22	- 11.11	33.33	25	- 8.33
**III degree (BMI >40 Kg/m** ^**2**^ **)**	42.86	28.57	- 14.29	22.23	11.11	- 11.12	58.34	41.67	−16.7

^1^All values are in percentage. BMI: Body Mass Index.

Anthropometric parameters, and body composition characteristics by DXA, at baseline for total population and according to MTHFR genotypes, are given in Table 4.

**Table 4:** **Anthropometric and body composition parameters at baseline and after HBD nutritional intervention according to genotypes** ^**1**^

**Parameters**	**Baseline**			**Week 12**		
	**Total (n = 44)**	**T (−) (n = 20)**	**T (+) (n = 24)**	**Total (n = 44)**	**T (−) (n = 20)**	**T (+) (n = 24)**
**Weight (Kg)**	97.79 ± 15.46	89.60 ± 14.43	103.93 ± 13.69^d^	88.17 ± 16.01^a^	80.51 ± 15.00^b^	93.92 ± 14.78^c^
**BMI (Kg/m** ^**2**^ **)**	40.72 ± 5.89	38.58 ± 7.23	42.32 ± 4.31	36.69 ± 6.05^a^	34.68 ± 7.38	38.20 ± 4.61^c^
**Abdomen (cm)**	124.43 ± 12.18	120.06 ± 12.76	127.71 ± 11.12	116.36 ± 12.59^a^	111.83 ± 12.54^b^	119.75 ± 12.02^c^
**Hip (cm)**	124.24 ± 10.94	119.22 ± 12.58	128.00 ± 8.17	116.55 ± 10.82	111.56 ± 12.09	120.29 ± 8.42^c^
**Neck (cm)**	41.93 ± 3.09	39.90 ± 2.88	42.74 ± 2.91	39.23 ± 2.90^a^	37.88 ± 2.36^b^	40.51 ± 2.98^c^
**TBFat (Kg)**	46.91 ± 8.88	41.03 ± 7.18	51.51 ± 7.57^d^	40.42 ± 9.36	35.21 ± 7.79^b^	45.29 ± 8.78^c,e^
**TBLean (Kg)**	47.67 ± 8.09	44.86 ± 6.96	50.17 ± 8.95	44.69 ± 7.26^a^	42.84 ± 6.61	46.33 ± 8.13^c^
**PBF (%)**	48.24 ± 3.52	46.50 ± 2.90	49.46 ± 3.56	45.87 ± 3.83^a^	43.57 ± 3.79	48.01 ± 2.78
**BMD total body (g/cm2)**	1.21 ± 0.12	1.15 ± 0.11	1.27 ± 0.11	1.19 ± 0.12^a^	1.14 ± 0.11	1.24 ± 0.11^c, e^
**BMC total body (g)**	2460.65 ± 351.30	2270.67 ± 255.15	2645.50 ± 355.62	2507.50 ± 408.15^a^	2270.67 ± 255.15	2750.60 ± 356.15^c^
**RMR (Kcal/die)**	1762.47 ± 299.00	1658.68 ± 257.43	1855.02 ± 330.95	1652.30 ± 268.55	1658.68 ± 257.43	1713.15 ± 300.66^c^

^1^All values are mean ± SD. BMI: Body Mass Index, TBFat: Total Body Fat; TBLean: Total Body Lean; PBF: Percentage of Total Body Fat; BMD: Bone Mineral Density; BMC:Bone Mineral Content; RMR: Resting Metabolic Rate.

^a^Reflects the significance of the differences between total population at baseline vs total population at week 12 determined with an independent t test (p ≤ 0.05).

^b^Reflects the significance of the differences between T(−) carrier at baseline vs T(−) carrier at week 12 determined with an independent t test (p ≤ 0.05).

^c^Reflects the significance of the differences between T(+) carrier at baseline vs T(+) carrier at week 12 determined with an independent t test (p ≤ 0.05).

^d^Reflects the significance of the differences between T(−) carrier at baseline vs T(+) carrier at baseline determined with an independent t test (p ≤ 0.05).

^e^Reflects the significance of the differences between T(−) carrier at week 12 vs T(+) carrier at week 12 determined with an independent t test (p ≤ 0.05).

After 12 weeks HBD nutritional intervention, significant decreases in body weight (p ≤ 0.05), BMI (p ≤ 0.001), waist and abdomen circumpherences (p ≤ 0.05), TBFat (p ≤ 0.05)and TBLean were observed in the total population.

Dietary treatment success was achieved by all subjects. Of total subjects, all decreased in terms of weight and TBFat: nobody increased weight.

At baseline, T(+) carriers had higher body weight, waist circumpherence, TBFat, TBLean, PBFat and BMD total body respect to T(−) carriers.

Table 5 shows the comparison of body composition relative changes (∆%) according to genotype.

**Table 5:** **Comparison of body composition relative changes according to genotype** ^**1**^

**∆%**	**Total (n = 44) (min; max)**	**T (−) carrier (n = 20) (min; max)**	**T (+) carrier (n = 24) (min; max)**
**∆% weight**	−10.09 ± 3.57 [−19.52; −5.77]	−10.42 ± 3.19 [15.54; −7.16]	−10.10 ± 3.95 [− 19.52; −5.77]
**∆% TBFat**	−13.52 ± 5.84 [−25.63; −3.17]	−14.70 ± 5.34 [−25.63; −8.53]	−12.45 ± 4.38* [−24.56; −3.17]
**∆% TBLean**	−6.06 ± 4.22 [−12.84; +2.66]	−4.46 ± 2.90° [−8.16; 0.44]	−7.51 ± 3.17* [−12.84; 2.66]

^1^All values are mean ± SD. TBFat: Total Body Fat; TBLean: Total Body Lean.

*Reflects the significance of the differences between T(−) carrier vs T(+) carrier at baseline and at week 12 determined with an independent t test (p ≤ 0.05).

°Reflects the significance of the differences between total population vs T(−) carrier at baseline and at week 12 determined with an independent t test (p ≤ 0.05).

Significant ∆% TBFat (p ≤ 0.05) were observed between T(−) carriers and T(+) carrier. Moreover, significant ∆% TBLean (p ≤ 0.05) were observed between total population and T(−) carriers and between T(−) carriers and T(+) carrier.

## Discussion

Globally, the prevalence of metabolic syndrome is increasing, largely because of obesity resulting from poor diets and sedentary lifestyles. Moreover, impaired glucose tolerance and diabetes mellitus may be found in nearly 20% of women age 55–65 years, and high blood glucose is globally the third leading cause of premature mortality [27]. Furthermore, studies showed that individuals with sarcopenic obesity had the highest morbidity and mortality [28], and that lean body mass exerts a protective effect against all cause mortality [29]. A dietary approach might significantly help prevent and treat the metabolic imbalance, and a correct life style is necessary to preserve health status and wellbeing. Lifestyle changes (LC) interventions, such as dietary changes and physical activity, have a main role in treatment of the MS [30,31].

However, it has long been suspected that “one size does not fit all” in terms of determining the optimal diet for an individual, and this has been demonstrated over the years in studies on gene-diet interactions and with the emergence of nutrigenetics [32].

It’s an emerging area that genetic variants could be considered in obesity and disease management for their additive effects [33-35].

Strategies to improve weight maintenance are nowday focused on considering together the genetic makeup and its interaction with dietary intake, with the aim to identify vulnerable populations/individuals that will benefit from a variety of more personalized and mechanistic-based dietary recommendations [36].

To the best of our knowledge, this is the first study to evaluate the impact of C677T MTHFR genetic variant on hysocaloric balanced nutritional intervention response in italian obese women affected by MS. This new approach, based on genetic and body composition evaluations, defines the requirements to be assessed as guidelines to identifying a really effective diet and could improve long term weight management.

Because of the association of C677T MTHFR polymorphism with cardiovascular disease risk, osteoporosis and sarcopenia [10,37,38], we considered the T(+) carriers as at risk subjects.

Our findings suggest that C677T MTHFR polymorphism may play a significant role in body and metabolic factors changes, induced by HBD. In particular, all subject responded to HBD in term of weight loss, independently by genotype. The prevalence of MS parameters decreased by 84% for blood pressure, by 79.5% for HDL-C, by 82% for fasting glucose, and by 77% for triglycerides.

At baseline, T(−) carrier showed a higher level of fasting glucose and a lower triglycerides level than T(+) carrier (p ≤ 0.05). During the observation period, the significant differences among the MS components occurred. According to the fact that, monounsaturated, and n-3 fatty acids present in the diet help to reduce BP levels, and risk of developing hypertension [39], in our total population diastolic and systolic BP levels was reduced (p ≤ 0.05). Moreover, T(−) carrier showed a lower SBP level (p ≤ 0.05). At baseline T(+) carrier showed an higher levels of waist circumference, Tryglicerides, and lower fasting glucose (p ≤ 0.05). The HBD was associated with reduction in waist circumference in total population (p ≤ 0.05), in T(−) and T(+) carriers respectively (p ≤ 0.05). Moreover, significant reduction of fasting glucose was observed in T(−) carrier, more than T(+) carriers (p ≤ 0.05).

Several authors have estimated that a 1% decrease in BMI across the United States population would prevent more than 2 million new cases of diabetes over the next 20 years [40]. Moreover, recent lines of evidence support the hypothesis regarding the heritability of body composition. Different genes may contribute to the expression of fat and lean mass. It has been well established that TBLean, TBFat, and BMI are under strong genetic control, with heritability of 0.52–0.72 [41].

Body composition analysis is a pivotal strength of our study. Measures of body fat and lean distribution, could help to stratify individuals according to their level of body fat and preserved lean mass and to reveal the real impact of a nutritional intervention [13].

In a recent study, [42] we have observed changes in terms of body composition in an Italian Caucasian obese population after 12 weeks of Italian Mediterranean Diet (IMD) based on C677T MTHFR gene polymorphism.

Before the nutritional intervention, we observed differences in body composition between MTHFR genotypes groups, indeed T(+) carriers were fatter than T(−) carriers.

Interestingly, the highest number of responders to IMD nutritional intervention with the lower TBLean (Kg) loss were T(−) carriers. T(+) carriers lost more lean mass, particularly at trunk level, and less fat mass, particularly at android level, than T(−) carriers.

The herein shown results demonstrate that 12 weeks HBD nutritional intervention is a valid dietary approach for loosing weight and particularly TBFat, in obese women. However, an inter-individual variability, a well-known phenomenon in nutrition research and practice, was highlighted. The mechanisms responsible for the inter-individual differences in dietary response are very complex and poorly understood. The concept of gene-diet interaction describes the modulation of the effect of a dietary component on a specific phenotype, such as obesity, by a genetic variant. A role of genetic factors contributing to those differences in response to nutrients has been proposed for several decades and successfully demonstrated for rare inborn metabolism errors [43].

However, it is unknown whether low compliance to dietary instructions, that plays an important role in body composition, may account for the differences in the response to nutritional interventions. It is important to note that at the beginning of dietary plan, subjects experienced the most dramatic weight loss, because they were very compliant to the administered diet. Thus, in our study the HBD was prolonged only for 12 weeks, in order to reveal the true impact of given genetic polymorphisms or their combination on weight loss and body composition changes after a nutritional intervention.

At baseline, we observed differences in body composition, and metabolic parameters between MTHFR genotypes groups. T(+) carriers were fatter than T(−) carriers, with higer body weight and TBFat (p ≤ 0.05).

Moreover, in the 81% of the total population a loss of Total Body Lean was observed (p ≤ 0.05).

A significative loss (p ≤ 0.05) of Total Body Lean was observed in the 47% of T(−) carriers and in the 53% of T(+) carriers.

In particular, after the HBD the presence of C677T polymorphism in the MTHFR gene is associated with a higher decrease of lean mass and lower reduction of fat mass. Interestingly, according to Δ% , the highest number of responders to HBD with the lower TBLean (Kg) loss were T(−) carriers. As proposed by Lambrinoudaki et al. [44], the genetic variants may lead to tissutal dysfunction, thus causing metabolic disturbances and related body composition modification.

Reduction in lean body mass per se is an important risk factor for sarcopenia, a serious health problem that has not yet received sufficient attention. Sarcopenia is a syndrome characterised by progressive and generalised loss of skeletal muscle mass and strength with a risk of adverse outcomes, including, but not limited, to impaired protein turnover, mobility loss, osteoporosis, dyslipidemia, insulin resistance, overall frailty, and increased mortality [45]. In the past decade, a number of candidate genes for obesity and osteoporosis have been identified [46,47], but the genetic basis of sarcopenia is still largely unknown. Sarcopenia is usally referred to the age-related decrease in muscle quantity, quality, and function. In elderly individuals, changes in body composition result in the prevalence of overweight and obesity combined with a loss of muscle mass and strength; this has recently been defined as sarcopenic obesity [48].

The combination of sarcopenia and obesity is an important public health problem that induces fragility in the elderly, and it is associated with functional limitations and increased mortality [49-52]. In order to avoid the risk of sarcopenic obesity in the eldery, and to prevent in the young population, the diagnosis of obesity requires the utilization of various methods, including body composition evaluation and a genetic approach.

In Italian males and females the relation with body composition and anthropometric parameters and resting metabolic rate was well described [53].

On the basis of these results, we investigated the effects of MTHFR polymorphism on resting metabolic rate of obese women, making a comparison between T(+) and T(−) carriers. At baseline, no significant difference of RMR was observed. However, the reduction of RMR, observed in T(+) carriers more than in T(−) carriers (p ≤ 0.05), is proportional to the decrease on lean mass, and weight loss. We conclude that the measurement of energy expenditure normalised to metabolically active mass should provide a tool to define hyper and hypo-metabolic state during weight loss.

Liu et al. [10] propose the hypothesis that MTHFR may exert its effect on lean body mass variation by regulating mitochondria function and cell apoptosis, which awaits further studies for validation.

Although biochemical studies are needed to confirm this observation and to elucidate the molecular mechanisms that underlie and explain the relationship between MTHFR, TBFat and TBLean, this study represents a pilot effort to explore the role of gene environment interaction in the body composition evaluation.

An important limitation of this study is the relatively small sample size. However, it was large enough to provide us adequate statistical power and the associations have reached statistical significance in this context of limited sample size. Therefore, it may be thought of as valid and reproducible in larger samples.

Advances in the fields of molecular biology, biochemistry, and genetics enhanced the study of nutrition, moving towards the realization of Predictive, Personalized, Preventive and Participatory (P4) medicine [54]. The changes in medicine will trigger the emergence of personalized nutrition, focuses on the integrated diagnosis, and treatment for the prevention of diseases. Our data provide the basis for personalized dietary recommendations based on the individual’s genetic makeup and information from other factors such as gender, age, body composition and RMR, in the context of negative energy balance, like hypocaloric nutritional intervention assigned to obese subjects.

A significant association was also found between protein consumption per mealtime and loss of muscle mass [55]. For this reason, protein intake should be considered when evaluating the multi-factorial loss of physical function in women [56].

Based on the results of this pilot study, we suggest a tailored dietary approach, particularly for T(+) carrier women, who experienced the most dramatic loss of TBLean, assuring an adequate selection of protein and omega-3 fatty acids-rich foods to preserve muscle mass and prevent sarcopenia. Moreover, in order to decrease hypertriglyceridemia in T(+) carrier women, we suggest to increase the omega-3 assumption (2–4 g/day).

In conclusion, our data provide evidence of interaction between C677T MTHFR gene polymorphism and the metabolic response to diet. This pilot study highlighted the importance of develop personalized dietary guidelines to reduce MS risk among women with different genetic background. As health care budgets become more restricted in all countries with the passage of time, and given the great expense involved in treating serious complications of obesity and MS, a large-scale comprehensive interventions, according to the vision of social medicine, for the prevention and control of these pathologies should be regarded as of public health priority. The efficacy of genetic tests to assess in advance the effectiveness of a dietary treatment is an undoubted advantage for the diet therapy approach to prevent complications, and reduce the social costs of a possible drug therapy [57].
